# Genetic and Phenotypic Characterisation of a *Saccharomyces cerevisiae* Population of ‘Merwah’ White Wine

**DOI:** 10.3390/microorganisms7110492

**Published:** 2019-10-26

**Authors:** Nadine Feghali, Warren Albertin, Edouard Tabet, Ziad Rizk, Angela Bianco, Giacomo Zara, Isabelle Masneuf-Pomarede, Marilena Budroni

**Affiliations:** 1 Department of Agricultural Science, University of Sassari, 07100 Sassari, Italy; feghalinadine@gmail.com (N.F.); chicca14.ab@gmail.com (A.B.); gzara@uniss.it (G.Z.); 2 UR Œnologie EA 4577, USC 1366 INRA, University of Bordeaux, Villenave d’Ornon, 33882 Bordeaux, France; Warren.albertin@u-bordeaux.fr (W.A.); isabelle.masneuf@agro-bordeaux.fr (I.M.-P.); 3 Faculty of Agricultural Sciences-CRFA, Lebanese University, Ghazir, Lebanon; edytabet@hotmail.com; 4 Lebanese Agricultural Research Institute (LARI), 90-1965 Fanar, Lebanon; Zrizk@lari.gov.lb

**Keywords:** native yeast, spontaneous fermentation, genotype–phenotype diversity, regional wine

## Abstract

The study of yeast biodiversity represents an important step in the preservation of the local heritage, and this work in particular has an innovative character since no further studies have investigated ‘Merwah’, one of the main grape varieties used in winemaking in Lebanon. To gain deeper knowledge of the genetic diversity and population structure of native *Saccharomyces cerevisiae* wine strains, 202 isolates were collected during spontaneous alcoholic fermentation of eight must/wine samples of cultivar ‘Merwah’, over two consecutive years (2016, 2017) in a traditional winery in Mount Lebanon (1400 m a.s.l.). The isolates were identified as *S. cerevisiae* on the basis of their morphology and preliminary sequence analysis of their internal transcribed spacer (ITS) PCR. They were then characterised at the strain level by interdelta PCR and genotyped using multiplex PCR reactions of 12 microsatellite markers. High genetic diversity was observed for the studied population. To select potential yeast starter strains from this population, micro-fermentations were carried out for 22 *S. cerevisiae* strains that were selected as representative of the ‘Merwah’ wine yeast population in order to determine their technological and oenological properties. Three indigenous yeast strains might represent candidates for pilot-scale fermentation in the winery, based on relevant features such as high fermentation vigour, low production of volatile acidity and H_2_S and low residual sugar content at the end of alcoholic fermentation.

## 1. Introduction

In recent years, many wineries have been using selected indigenous yeast strains, which might persist more easily and dominate over other yeast strains. The inoculation of fermentation with autochthonous *Saccharomyces cerevisiae* strains provides distinctive characteristics to the wine and helps to preserve the native yeast strains that are better adapted to the environment of the viticulture region and to the winemaking process [1,2]. In addition, *S. cerevisiae* strains identified in different wineries in the same wine region are considered to be representative of an oenological zone [3].

Several studies have shown that grape varieties, geographic location, climate conditions, the chemical composition of the initial must, biotic factors (e.g., microorganisms, killer factors, migratory birds, grape varieties), abiotic factors (e.g., pH, temperature, ethanol, osmotic pressure, nitrogen, molecular sulphur dioxide) and anthropogenic factors (e.g., use of commercial starters) can affect the diversity of an *S. cerevisiae* population [4,5,6,7,8,9,10,11].

Lebanon is at the eastern extremity of the Mediterranean and is one of the oldest of the countries of the Old World. The land is particularly favourable for the cultivation of grapevines, which are among the oldest cultivated [12]. Lebanon has a rich heritage of native grape varieties, such as ‘Merwah’, ‘Obeidi’, ‘Tfeifihi’, ‘Beitamouni’ and ‘Maghdouchi’. Of these, the main local grape varieties used in winemaking are ‘Merwah’ and ‘Obeidi’, which have attracted the attention of wine consumers [13]. The presence of such indigenous grape varieties allows the determination of the genetic map of Lebanon. To date, and to the best of our knowledge, no previous studies have investigated ‘Merwah’ wines. Therefore, in the present study, the genetic and phenotypic characteristics of Lebanese indigenous *S. cerevisiae* wine yeast were evaluated for yeast ecology during spontaneous fermentation, and the indigenous yeast were screened for use as potential starters for the production of wines with regional characteristics.

The characterisation of the autochthonous *S. cerevisiae* strains is an important step towards the conservation and exploitation of microbial biodiversity. The aim was therefore to evaluate the genetic diversity and relatedness among *Saccharomyces cerevisiae* strains isolated from ‘Merwah’ wine, in order to select starter strains that be useful in Lebanese wine production. For this objective, 202 isolates were collected from eight must/wine samples of ‘Merwah’ grapes during spontaneous alcoholic fermentation over two consecutive years (2016, 2017), from the ‘Château Bybline’ Lebanese winery.

Genetic characterisation was performed using PCR amplification of the interdelta sequences. In this large population of isolates, 22 yeast strains were selected as representative of the major strain clusters of the interdelta sequence profiles. The identification of *S. cerevisiae* was confirmed by PCR–restriction fragment length polymorphism of the 5.8S internal transcribed spacer (ITS) rDNA, with all of the 202 isolates confirmed as *S. cerevisiae* [14]. In addition, the yeast isolates were genotyped for 12 microsatellite markers to provide a deeper analysis of their genetic diversity and the population structure.

The technological characterisation of these yeast were evaluated in synthetic grape juice (SGJ) at the laboratory scale, according to the main parameters (i.e., residual sugar, fermentative vigour and kinetics, lowest production of sulphur products (H_2_S, SO_2_), acetic acid) in order to select the *S. cerevisiae* strains that might be used as starter cultures at an industrial scale.

## 2. Materials and Methods

### 2.1. Winery and Winemaking Process

This study was carried out at the ‘Château Bybline’ winery, where the ‘Merwah’ autochthonous white grape variety is planted around the estate (altitude, 1400 m a.s.l.). ‘Merwah’ grapes were harvested from two vineyards at Wata al Jawz (2000 m^2^) and Bekaatet Achkout (2000 m^2^), in Keserwan, Mount Lebanon region. This winery has been using traditional viticulture practices with old grapevines (for >100 years), with low yields (<30 hL/ha) and spontaneous fermentation.

The harvesting season was planned according to the acidity and sugar concentration of the grapes in 2016 and 2017. Before the grape pressing, the winemaker used ‘skin maceration’, where the crushed grapes were left in a vat as a macerate for a few hours, to extract the maximum aromas from the grapes. Sulphite was added as a gassed liquid solution of SO_2_ during grape pressing (5 g/hL), and after the malolactic fermentation and racking (5 g/hL). ‘Merwah’ musts were fermented in French oak wood barrel during the process of fermentation. The temperature of the alcoholic fermentation varied from 15 to 18 °C.

### 2.2. Sample Collection, Isolation and Morphological Identification

Eight of the ‘Merwah’ must/wine samples were collected at the middle and final stages of their spontaneous alcoholic fermentation. Chemical analysis of the must was carried out to determine the density, sugar content, pH and total acidity during alcoholic fermentation. Yeast isolation and morphological identification was performed after thawing must/wine samples previously collected and maintained at −18 °C with added 75% glycerol.

The samples were inoculated onto YPD agar (1% yeast extract, 2% peptone, 2% glucose, 2% Bacto-agar) and incubated at 25 °C for 48 h. The colonies were sampled and cultivated on fresh YPD agar. Then replica plating was carried out on differential Wallerstein Laboratory Nutrient media (WL media) at 25 °C for 2 days [15].

According to the colony morphologies and colours on WL agar and under light microscopy, 202 isolates were identified as *S. cerevisiae*. These isolates were maintained as frozen stocks (40% glycerol, *v*/*v*) as static culture in YPD liquid medium at −80 °C before use and are stored in the University of Sassari (Italy) strain collection. Twenty-two of these yeast strains were selected from this large population of isolates as representative of the major strain clusters of the interdelta sequence profiles [14]. This technique allows discrimination of the *S. cerevisiae* isolates, as no amplicon is generated for other *Saccharomyces* and non-*Saccharomyces* species [16]. Identification of the 22 *S. cerevisiae* isolates was confirmed by PCR of the internal transcribed spacer (ITS) region of ribosomal DNA [17].

### 2.3. DNA Extraction

The yeast isolates were cultivated overnight in YPD liquid at 25 °C. Then 1.5 mL samples of the cell biomass were centrifuged at 13,000× *g* for 5 s, with the supernatants eliminated. The pellets were resuspended in 200 μL extraction mix: (2% Triton 100×, 1% sodium dodecyl sulphate, 100 mM NaCl, 10 mM Tris-HCl, 1 mM Na_2_EDTA, phenol: chloroform: isoamyl alcohol (25:24:1)) with 0.3 g 212–300-μm-diameter glass beads (Sigma-Aldrich, St Louis, MO, USA). The samples were vortexed for 2 min and then centrifuged at 13,000× *g* for 5 min. The DNA was precipitated from the supernatants by adding three vol. 100% ethanol and 0.1 vol. 3 M NaOH, with the samples cooled to −80 °C for 20 min. The samples were then centrifuged at 13,000× *g* for 15 min at 5 °C. The pellets were washed with 70% ethanol and centrifuged at 13,000× *g* for 15 min, and then vacuum dried. The DNA extracted was dried and suspended in 50 μL TE buffer (0.1 M Tris, 0.1 M EDTA, pH 8.0) and stored at −20 °C [18]. The efficiency of this DNA extraction procedure, and its purity and concentration, were measured using a spectrophotometer (NanoDrop; BMG Labtech, Offenburg, Germany) [19].

### 2.4. Interdelta PCR Typing

The DNA suspensions (50 ng/μL) were added to reaction mixtures (25 μL) that contained: Taq buffer (+1.5 mM Mg^2+^) (1×), 25 mM MgCl_2_, 0.2 mM primer 1 δ2 (5′-GTGGATTTTTATTCCAAC-3′), 0.2 mM primer 2 δ12 (5′-TCAACAATGGAATCCCAAC-3′) [20], 0.2 mM dNTP and 1 U Taq polymerase (Trans Gene Biotech, Beijing, China). Amplification reactions were performed on a PCR machine (Thermal Cycler T-100; BioRad, Milan, Italy) using the following programme: 95 °C for 30 s, 52 °C for 30 s, 72 °C for 90 s, and final extension at 72 °C for 10 min.

The products of the PCR reactions were analysed by electrophoresis on 1.5% agarose gels, which were then stained with SYBR safe and visualised under a UV transilluminator (Chemi Doc XRS imaging system; BioRad, Milan, Italy). All of the visible bands were assigned a number based upon their relative position in the DNA ladder. Each position was then assigned ‘0’ or ‘1’ to indicate the absence or presence of the band, respectively. The 0/1 matrix was then used to generate the dendrograms. The comparative cluster analysis of different strains integrated the banding pattern data over the two consecutive years (i.e., 2016, 2017).

### 2.5. ITS-PCR

Identification of the selected yeast was performed by amplification and sequencing of the ribosomal DNA ITS region. The DNA suspensions (50 ng/μL) were added to the PCR mixture (final volume, 50 μL) that included 1× buffer, 0.2 mM dNTP, 1.5 mM MgCl_2_, 0.5 μM primers ITS1 (5′-TCCGTAGGTGAACCTGCGG-3′) and ITS4 (5′-TCCTCCGCTTATTGATATGC-3′), and 1 U Taq polymerase [17]. The reaction involved initial denaturation at 95 °C for 10 min, followed by 30 cycles of the series of 95 °C for 1 min, 55 °C for 1 min, and 72 °C for 90 s, with a final cycle at 72 °C for 10 min.

The amplicons were purified using PCR purification kits (QIAquick; Qiagen GmbH, Hilden, Germany), following the manufacturer instructions, and were sequenced by the BMR Genomics Laboratory (www.bmr-genomics.it; Padova, Italy). The sequences obtained were compared with those in the GeneBank database (NCBI) using the BLAST programme [21]. Sequences with ≥97% identity were considered to represent the same species.

### 2.6. Microsatellite Amplification

Each DNA sample was diluted and adjusted to 50 ng/μL DNA and genotyped using two multiplex PCRs of five and seven microsatellite loci for mixtures #1 and #2, respectively (Table 1). For each PCR reaction, a total volume of 1.9 μL was taken from each mix, then 6.25 μL Qiagen multiplex PCR master mix (2×) was added to 1 μL DNA, with MilliQ water added to take the final volume to 12.5 μL. The amplification reactions were performed on a PCR machine (Thermal Cycler T-100; BioRad, USA) using the following programme: initial denaturation at 95 °C for 15 min, followed by 35 cycles of 95 °C for 30 s, 57 °C for 2 min and 72 °C for 1 min, and final extension at 60 °C for 30 min.

PCR product sizes were obtained for 12 microsatellite loci on a capillary DNA sequencer (ABI 3730xl DNA analyser; Applied Biosystem; Singapore, Republic of Singapore) with DS-33 Matrix Standard kits using the polyacrylamide Pop 7, and the size standard 600LIZ (GeneScan 600 LIZ Size Standard v2.0; Thermo Fisher, San Diego, CA, USA). Raw sizes were assigned into classes of alleles of similar size using GeneMarker version 2.6.3 (Demo). The sizes of microsatellite amplicons were used to investigate the genetic relationships between the strains.

After microsatellite typing, the presence of missing values was authorised to a maximum of three markers per sample, and they were taken into account in the analyses considering that they might reflect part of the diversity. Only individuals with more than 5/12 microsatellite data were kept in the end, 194 yeast strains were genotyped according to the 12 microsatellite markers.

The recorded allele sizes for the 12 microsatellite markers were analysed using the R software V3.2.5. [26]. A dendrogram was constructed using Bruvo’s distances [27] and neighbour-joining clustering [28], with the *poppr* (V2.8.0) [29,30] and *ape* (V3.2.5.) [31] packages. Multidimensional scaling was performed with Bruvo’s distance matrix, with the *cmd scale* function in R. As this analysis does not allow for missing data, the nearest neighbour method (on the basis of Bruvo’s distances) was applied to impute the missing data.

Population structure analysis was performed using the package LEA built under R V3.2.5. [32] and the non-negative matrix factorisation algorithm [33] to estimate individual ancestry coefficients. Models with populations (*K*) from 1 to 45 were tested in 100 repetitions.

In order to compare the studied population to other population origins, 138 *S. cerevisiae* strains from different origins (i.e., bioprocess, wild, wine strains), described in Table S4 in supplementary data, were added to this study.

The differentiation among the different yeast populations (i.e., bioprocess, wild, wine, Lebanon ‘Merwah’ wine strains) was analysed through the fixation index (*Fst*), computed with the polysat package [34]. Here, 100 bootstraps were computed, and the confidence intervals were calculated for the *Fst* indices.

Diversity indices were calculated using the *poppr* package [29,30], with Simpson’s index and Shannon’s equitability index (e.g., Shannon’s index taking into account the population size). The Shannon index is calculated as follows: *H*’ = −∑*p_i_*·ln*p_i_*; where *p_i_* is the proportion of individuals in species *i*. For a well-sampled community, we can estimate this proportion as *p_i_* = *n_i_*/*N*, where *n_i_* is the number of individuals in species *i* and *N* is the total number of individuals in the community. As by definition *p_i_*_s_ will all be between zero and one, the natural log makes all of the terms of the summation negative, which is why we take the inverse of the sum. Typical values are generally between 1.5 and 3.5 in most ecological studies, and the index is rarely >4. The Shannon index increases as both the richness and the evenness of the community increase. The Simpson index is based on the probability of any two individuals drawn at random from an infinitely large community belonging to the same species: *D* = ∑*p_i_*^2^; where again *p_i_* is the proportion of individuals in species *i*. For a finite community, this is: *D* = ∑*n_i_*(*n_i_* − 1)/*N*(*N* − 1). As species richness and evenness increase, diversity also increases. The values of *D* lie between 0 and 1.

### 2.7. Microfermentation and Phenotypic Analysis

After genetic characterisation, 22 *S. cerevisiae* strains were selected based on the main clusters obtained from the interdelta PCR results and the ancestral clustering of microsatellites analysis. To select yeast strain(s) with interesting fermentative performance and technological features, oenological characterisation was carried out for the 22 selected strains (Table 2).

The yeast strains were pre-cultured in 100 mL synthetic media (50 g/L glucose, 1 g/L yeast extract, 2 g/L (NH4)_2_SO_4_, 0.3 g/L citric acid, 5 g/L l-malic acid, 5 g/L l-tartaric acid, 0.4 g/L MgSO_4_, 5 g/L KH_2_PO_4_) [35] for 2 days at 22 °C in an incubator-shaker at 250 rpm (Multi Stack, shaking; LabTech, Sorisole (BG), Italy). To evaluate the strain-specific fermentation performances, micro fermentations were carried out by inoculation of each yeast strain in triplicate to the final concentration of 3 × 10^6^ cell/mL in 250 mL synthetic grape juice (SGJ) (100 g/L glucose, 100 g/L fructose, 1 g/L yeast extract, 2 g/L (NH_4_)_2_SO_4_, 0.3 g/L citric acid, 5 g/L l-malic acid, 5 g/L l-tartaric acid, 0.4 g/L MgSO_4_, 5 g/L KH_2_PO_4_) [35]. The flasks were locked with Müller valves to allow only the CO_2_ to escape from the system and incubated at 22 °C in the incubator-shaker at 250 rpm (Multi Stack, shaking; LabTech) for all of the alcoholic fermentations.

The kinetics of the fermentations were monitored daily using gravimetric determinations to evaluate the loss of weight due to the production of CO_2_ (g/100 mL) [36]. In addition, daily measurements of the cell concentrations (cells/mL), OD 600 nm and biomass (g/L) were carried out [37]. Furthermore, the biochemical parameters were determined at the end of the alcoholic fermentation to select the pertinent strain(s) for the technological properties for wine fermentation. Several parameters were evaluated as follow:

- H_2_S production on BiGGY agar (Bismuth Sulphite Glucose Glycine Yeast, Difco, Sparks (MD), USA). The quantities of H_2_S produced by the yeast strains were evaluated qualitatively by colony colour formation, with scoring of the degree of browning (1–6) associated with the yeast growth, according to the following scale: white, 1; cream, 2; light brown, 3; brown, 4; dark-brown, 5; black, 6 [38].
- CO_2_ production (g/100mL) using the gravimetric method [36].
- Volatile acidity, using enzymatic reaction kits (Cat. No. 10148261035; Boehringer Mannheim, R-Biopharm, Darmstadt, Germany) with a double-beam UV/Vis spectrophotometer (UV S100; Shimadzu, Duisburg, Germany) ), and expressed as g/L acetic acid.
- Residual sugar by UV-visible spectrophotometry, with the dinitrosalicylic acid method, and expressed as g/L [39].
- Ethanol concentration by considering the theoretical yeast yield ~16.83 g/L to produce 1% alcohol: (initial sugar concentration—Residual sugar)/16.83 [40] and expressed as %.
- Total and free SO_2_ using the modified Ripper iodometric method and expressed as mg/L [41].
- Total acidity, with titration using 0.1 M NaOH to pH 7.00 ± 0.05, with the concentration determined here by acid-base titration and expressed as g/L sulphuric acid.
- The pH, with a pH meter (ST3000; Ohaus Co., Parsippany, NJ, USA).

### 2.8. Statistical Analysis of the Micro-Fermentations

All of the experimental measurements were conducted in triplicates. Means and standard deviations of the assays were calculated using conventional statistical methods. The ability of CO_2_ production at the end of the fermentation was analysed statistically using ANOVA followed by Tukey HSD test (*p* < 0.01). The relationships among yeast strains and their qualitative and quantitative phenotypic characteristics (fermentation vigour, ethanol production, H_2_S production, free and total SO_2_ production, volatile acidity, total acidity, pH and residual sugar) structured into groups, were summarized and visualized using Multiple factor analysis (MFA) (R package ‘FactoMineR’).

## 3. Results

### 3.1. Genetic Characterisation

A total of 112 and 90 isolates were collected from spontaneous alcoholic fermentations during the two consecutive years of 2016 and 2017, respectively, at the middle and the end of the alcoholic fermentations (Table 3). The isolates were initially classified as *S. cerevisiae* based on colony morphology on WL nutrient media.

#### 3.1.1. Genotyping by Interdelta PCR

The interdelta sequence patterns obtained after the gel electrophoresis were used to cluster the 202 yeasts isolates. As a first step, all of the isolates (112 from 2016, 90 from 2017) were subjected to cluster analysis together to test for similarities between the isolates from one year to the other. This analysis highlighted three main groups according to the year of isolation (one cluster for 2016, two clusters for 2017; Table S1). No genetic similarities were seen between the yeast isolates from 2016 and 2017.

**Figure 1:**
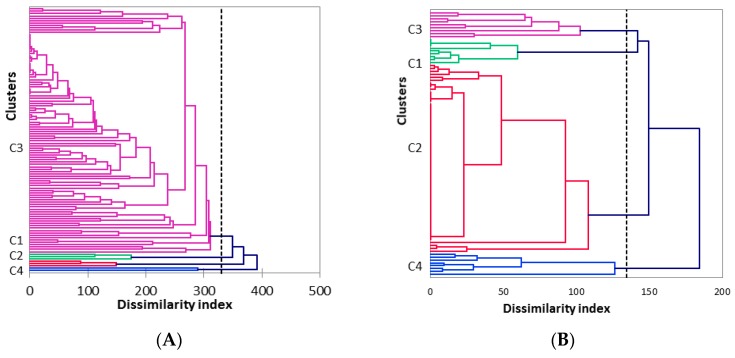
Dendrogram of the agglomerative hierarchical clustering (AHC) of the interdelta PCR for the 112 and 90 yeast isolates from 2016 (**A**) and 2017 (**B**), respectively. The dissimilarity is based on Euclidean distances (pbarret.net, 2005), and clusters are shown in four colours (C1: green; C2: red; C3: purple and C4: blue). In 2016, some of the isolates of the first batch were separated from the others (three isolates from the middle (2016-1MF) and five from the end (2016-1EF) of the alcoholic fermentation). For 2017, the isolates from the first and second batches were mixed in four clusters. Heavy blue line represents the relevant clusters considered. The dotted line corresponds to the minimum dissimilarity level considered in the analysis in order to define the relevant cluster.

For this reason, further cluster analysis was carried out separately for the isolates of each harvesting season. The dendrograms show the genetic dissimilarities of the various *S. cerevisiae* strains identified for both seasons (2016, 2017) (Figure 1). On the basis of agglomerative hierarchical clustering, four major clusters were identified for each season. High dissimilarities were seen across the isolates of 2016 and 2017 for the same cluster number (cluster 4; Tables S2 and S3, for 2016, 2017, respectively). It should be noted that the degree of dissimilarity in 2016 was twice that in 2017, which means that for 2016, higher differentiation was seen between the isolates, with greater similarity among the isolates in 2017.

From the initial 202 isolates, 22 of the yeast strains that were representative of the main clusters after the interdelta sequence profiles of 2016 and 2017 were confirmed as *S. cerevisiae* after sequencing of their ITS rDNA. Therefore, and according to previous studies, all 202 isolates can be considered as *S. cerevisiae* [14].

#### 3.1.2. Biodiversity of *S. cerevisiae* Strains According to Microsatellite Markers

Among the 202 *S. cerevisiae* isolates, 194 autochthonous isolates were analysed at 12 microsatellite loci (it was not possible to cultivate eight of the isolates), to study their genetic diversity and population structure. Two diversity indices that used the *poppr* package [29,30] were evaluated: Shannon’s equitability index (*H*’, also termed the Shannon-Wiener index), which measures the diversity within a population and takes into account the population size; and the alternate of Simpson’s index of diversity (1-*D*) that is used to compare diversity among communities and gives more weight to common or similar species [42]. These different indices were evaluated on the basis of the number of different genotypes and the standard deviations of *H*’ and *D*.

**Figure 2:**
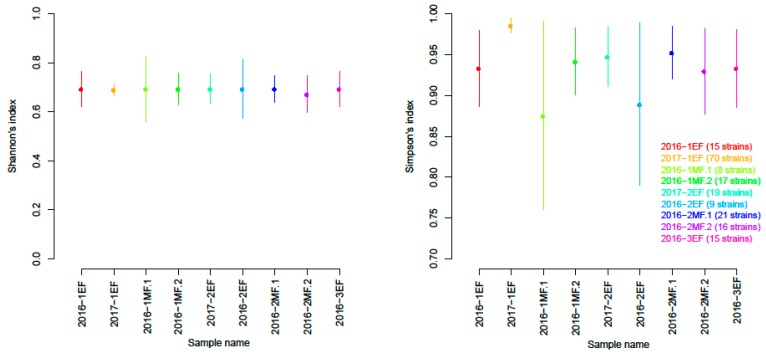
Shannon’s equitability and Simpson’s diversity indices for the *S. cerevisiae* isolates during the ‘Merwah’ winemaking. The indices were calculated using the *poppr* package.

Only samples with more than five isolates were considered for this analysis. The Shannon index was high and similar, regardless of sample or fermentation stage, thus showing high diversity in the Lebanese ‘Merwah’ yeast population. In addition, the inverse Simpson index was high (>0.98; Figure 2).

From the initial 194 Lebanese *S. cerevisiae* isolates, 180 different genotypes were identified, thus revealing high genetic diversity in the fermentations studied. To investigate the relationships between these ‘Merwah’ wine strains and those from other origins, a total of 138 strains of *S. cerevisiae* isolated from different sources were added to this analysis (e.g., from bioprocess, wild, wine industrial strains; Table S4), using the data from the 12 microsatellite markers [43]. Due to the clonal individual genotypes during fermentation, it was necessary to remove identical genotypes from the sample.

**Figure 3:**
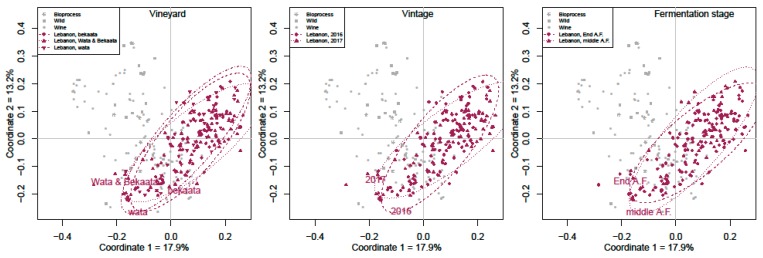
The MULTIDIMSCALE factors. Multidimensional scaling of the Lebanon ‘Merwah’ *S. cerevisiae* wine strains according to vineyard, vintage and fermentation stage. Dotted circle regroup strains population refer to their factorial group.

The microsatellite patterns for the indigenous ‘Merwah’ wine yeast were different from those of the *S. cerevisiae* strains of the industrial wines. Multidimensional scaling analysis showed that the vineyard origin, vintage and fermentation stage had no significant impacts on the genetic diversity of these Lebanese *S. cerevisiae* strains (Figure 3).

**Figure 4:**
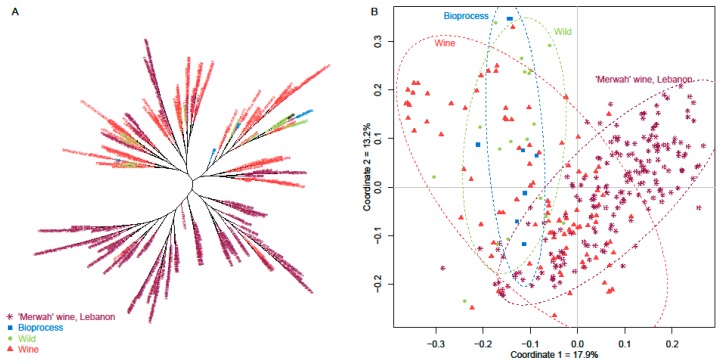
TREE-MULTIDIMSCALE. Population analysis of the 334 *S. cerevisiae* strains using 12 microsatellites. (**A**) Dendrogram using Bruvo’s distance and neighbour-joining clustering. (**B**) Multidimensional scaling. Coordinates 1 and 2 explain 17.9% and 13.2% of the variation. The strains from the different isolations are represented by different colours: bioprocess strains (blue), wild strains (green), wine strains (red) and Lebanon ‘Merwah’ wine strains (purple).

To examine the genetic linkage between all of the genotyped *S. cerevisiae* isolates (194 yeast isolates from ‘Merwah’ wine, and 134 from other origins), a dendrogram was constructed using Bruvo’s distance and a clustering neighbour-joining tree (Figure 4A). The results show differentiation among the yeast strains isolated from the different sources (i.e., single nucleotide polymorphisms) and good distribution of these ‘Merwah’ wine yeast throughout the tree. Also, as shown in Figure 4B, some of the ‘Merwah’ wine yeast isolates were more coordinated to ‘wine’ strains. In another part, a group of ‘Merwah’ wine yeast were greatly differentiated from other population origins (i.e., bioprocess, wild, and wine strains).

To better compare the populations of the ‘Merwah’ wine yeast with the other populations (i.e., bioprocess, wild, wine strains), *Fst* statistics were calculated through the fixation index (*Fst*) (Table 4). Pairwise *Fst* indicated low and moderate differentiation between the Lebanon ‘Merwah’ wine strains and the bioprocess, wild and wine strains, respectively.

**Figure 5:**
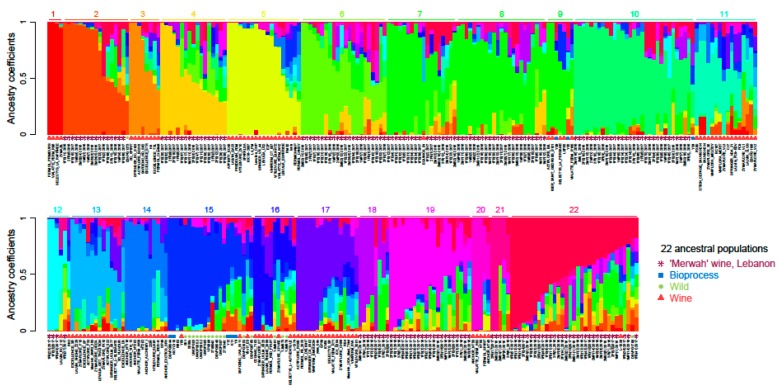
Barplots showing the population structure from the shared ancestry for the optimal *K* = 22. The population structure inferred using ADMIXTURE analysis for 332 populations and differentiated by four colours: purple, Lebanon ‘Merwah’ wine strains; blue, bioprocess strains; green, wild strains; red, wine strains.

The population structure from shared ancestry was evaluated for the 332 individuals (194 Lebanon ‘Merwah’ wine strains; 138 from other population origins: bioprocess, wild and wine strains, including industrial strains). The model with 22 ancestral populations (Figure 5) was the one with the lowest cross-entropy, as determined using Kruskal–Wallis tests (α = 0.05; package agricolae, V1.2-8) [45].

The ‘Merwah’ wine strains were assigned to 11 ancestral clusters, with numerous isolates composed of mosaic ancestral subpopulations. Ancestry profile analysis provided evidence that the ‘Merwah’ wine population and the wine strains are related.

### 3.2. Technological Characterisation

#### 3.2.1. Kinetics of the 22 ‘Merwah’ Wine *S. cerevisiae* during Alcoholic Fermentation in Synthetic Grape Juice

**Figure 6:**
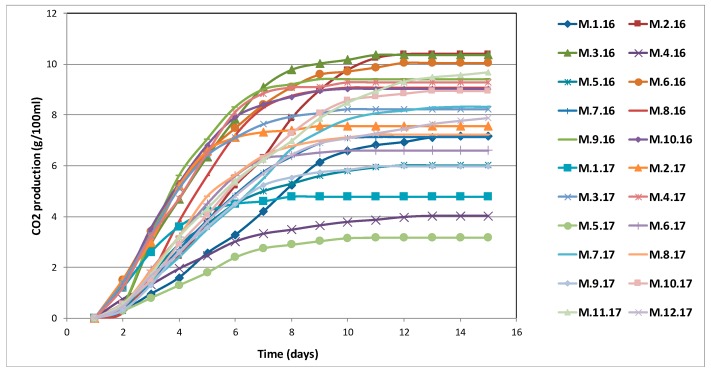
Carbon dioxide production by the 22 Lebanese *S. cerevisiae* isolates during alcoholic fermentation in synthetic grape juice (SGJ).

The fermentation kinetics of the ‘Merwah’ wine yeast strains were determined in triplicate by following their production of CO_2_ during fermentation in SGJ—Where not visible, error bars lie under the strain symbols (Figure 6). The ability of CO_2_ production was analysed through ANOVA followed by Tukey HSD test only at the end of the fermentation. Results are shown in Table S5 (as supplementary data).

These strains underwent fermentations over variable periods of 9 days to 15 days at 22 °C, and released high levels of CO_2_, which indicated their significant metabolic activities. Strain M.5.17 showed the lowest production of CO_2_. In contrast, nine strains showed a high production of CO_2_ (>8.9 g/100 mL): M.2.16, M.3.16, M.6.16, M.8.16, M.9.16, M.10.16, M.4.17, M.10.17 and M.11.17.

#### 3.2.2. Phenotypic Analysis

Twenty-two *S. cerevisiae* strains were fermented in the SGJ at 22 °C in order to evaluate the relevant parameters: fermentation vigour, ethanol production, H_2_S production, free and total SO_2_ production, volatile acidity, total acidity, pH and residual sugar (Table S6). Twelve of these 22 yeast strains consumed all of the sugar, as seen by their very low residual sugar (<2 g/L, whereas the others indicate incomplete fermentation. Among these six indigenous strains were low producers of H_2_S (M.3.16, M.6.16, M.4.17, M.10.17, M.11.17), which is an undesired compound during fermentation as it can confer unpleasant off flavours to the wine. This avoids yeast using their own amino acids (which contain the sulphur molecule) as a source of nitrogen [46].

Volatile acidity ranged from 0.01 to 0.69 g/L acetic acid. These concentrations are low and acceptable from an oenological point of view, and also according to European legislation (<0.9 g/L acetic acid) [47]. High variability was seen for the levels of SO_2_ produced by the 22 strains studied. At the end of the fermentation, these yeast strains produced ethanol from 11.10 to 11.88%. The final pH of the SGJ after fermentation was 3.2 to 3.5. Usually, low final pH (~3.1) can be explained by the release of organic acids by the strains during alcoholic fermentation, but this was not the case here. Indeed, acetic acid combined with ethanol can affect yeast fermentative behaviour, by decreasing the cell pH and the fermentation rate [48].

Finally, the fermentation vigour of these tested yeast strains was from 0.8 g CO_2_/L (M.5.17) to 3.53 g CO_2_/L (M.10.16). The fermentation vigour defines the speed at which the yeast starts their fermentation. This is an important criterion for dominance by starter yeast [48]. The most vigorous yeast strains are selected as dominant strains during wine fermentation.

The phenotypic data were evaluated by multiple factor analysis, with the aim to study correlations among groups of variables and how these can influence the co-variation among the different strains.

**Figure 7:**
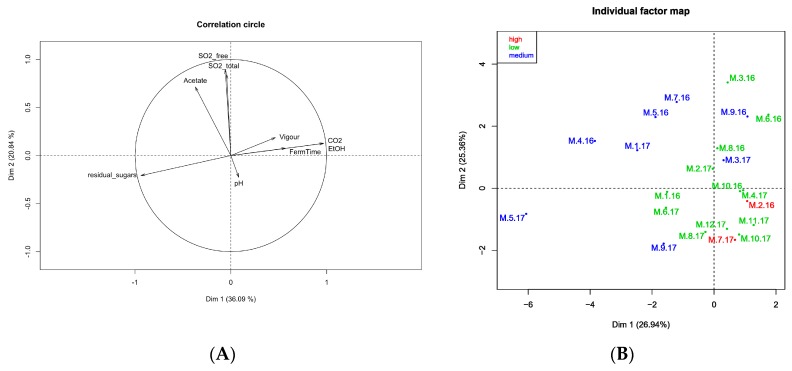
Correlation plots. (**A**) Relationship between the quantitative variables. (**B**) Individual factor map evaluating the dispersion of the yeast strains depending on the qualitative variable of ‘H_2_S production’.

Figure 7A shows the weight (or contribution) of each variable to the two axes. The degree of variability explained by the X axis and the Y axis was 26.94% and 25.36%, respectively. Then Figure 7B shows the positions of the strains on the axes, where their colours represent H_2_S production. The strains in green in Figure 7B are those that produced the lowest levels of H_2_S, which is recommended in the selection of wine yeast strains as starter cultures. The two yeast strains shown in red in Figure 7B (M.2.16, M.7.17) show high production of H_2_S. A high sulphite producer will not be chosen for starter culture due to the risk of off-flavour production during alcoholic fermentation.

The dispersion of these strains in the individual factor map (Figure 7B) shows the heterogeneity of these phenotypes. Among these strains, only one, which is far from the others, showed high residual sugar (M.5.17). This strain was thus eliminated as an unacceptable yeast starter for wine production, due to the risk of sluggish or incomplete fermentation.

Three strains, namely M.6.16, M.10.16 and M.4.17, may be selected as starters for microvinification assays as they showed the lowest residual sugar content at the end of alcoholic fermentation, the lowest production of volatile acidity and H_2_S, and the higher fermentation vigour.

## 4. Discussion

In this study, we analysed a population of *S. cerevisiae* yeast from the spontaneous fermentation of ‘Merwah’ wine to select for suitable potential autochthonous starter cultures for the improvement of the oenological production of this typical regional wine. At the end of fermentation, none of the isolate samples here belonged to non-*Saccharomyces* species, which will be due to the high ethanol in, and SO_2_ additions to, these wines [2].

High diversity across these *S. cerevisiae* strains was identified in the interdelta PCR analysis. This dissimilarity explains the absence of resident yeast from one year to the next and reveals the high genetic diversity within the populations from the same year. It is known that a low dominance of a certain species/strain can generate high diversity [16]. It has also been demonstrated that *S. cerevisiae* strains isolated during spontaneous fermentations from the same wine can be genetically distinct [49].

Many surveys have demonstrated high genetic diversity within the *S. cerevisiae* species that carry out spontaneous fermentations in different wine-producing regions [5,50]. These suggest that specific native *S. cerevisiae* strains might be associated with a given *terroir*, and that they can have an influence on the *terroir*-associated wine characteristics [3].

In addition, microsatellite marker analysis was used to determine the genetic linkage between these isolates, the ancestral alleles, and the population genetic structure. In this study, multilocus microsatellite analysis was performed with 12 loci, which allowed the evaluation of the genetic diversity among the Lebanese strains and between the Lebanese strains and strains of other origins. A total of 180 genotypes were revealed from an initial population of 194 *S. cerevisiae* isolates (eight of the strains among the 202 isolates had missing data) from this Lebanon ‘Merwah’ white wine, thus confirming high genetic diversity of the native *S. cerevisiae* throughout the alcoholic fermentation. These strains were different each year, and very few common strains were detected across these years, which indicate that there were no resident strains in this winery over the 2 years of the study.

The data obtained by the microsatellite analysis showed that this spontaneous wine fermentation is not driven by commercial isolates, but by a diversity of natural isolates. In the region studied, low dissemination of commercial strains is associated with high autochthonous genotype diversity, as also shown in a previous study [51]. However, based on the results of multidimensional scaling analysis, the diversity of the Lebanese yeast populations was not impacted by the vineyard location, production year or fermentation stage factors. Otherwise, geographic locations and ecological niches are both believed to have significant roles in *Saccharomyces* strain diversity [11,14].

Some wild yeast strains of Lebanon ‘Merwah’ wine clustered with the wine strains (including the industrial wine strains). This similarity may be explained by the fact that the winemaker received red grapes from other regions. For this reason, the oenological practices applied in the winery and the distribution of yeast populations from other winery areas or surrounding vineyard regions might affect the yeast diversity [52,53,54,55]. Also, the other part of the Lebanon ‘Merwah’ wine strain population was differentiated from the populations with other origins and formed an independent group. This differentiation reveals that these indigenous Lebanese yeast strains of the autochthonous ‘Merwah’ grape must have a different origin. This supports the concept that indigenous yeast strains selected in a winery can be associated with a *“terroir”*, and thus reflect the biodiversity of a particular area [56,57]. It has been demonstrated that genetic characterisation of wide groups of *S. cerevisiae* strains from different geographic origins and technological groups are related to their ecologically important phenotypic traits [51].

Based on the data from cluster analysis of interdelta PCR and the data obtained in ancestral analysis (11 ancestries for the Lebanon ‘Merwah’ wine yeast strains), 22 *S. cerevisiae* strains were defined as representative of this ‘Merwah’ wine yeast population, and these were selected for phenotypic characterisation. The strains characterised by molecular analysis were also used in micro-fermentation assays to evaluate their technological and oenological properties, with the aim of defining a starter culture. The selection of these yeast isolates was performed according to their characteristics: first, low production of H_2_S during fermentation, as H_2_S is highly undesirable and produces a rotten eggs odour, and then, low production of volatile acidity produced at the end of fermentation (as expressed by g/L acetic acid: <0.7 g/L), which can also negatively affect the wine aroma [2]. The acetic acid is affected by the addition of SO_2_ (i.e., less acetic acid is produced when SO_2_ is added), the turbidity (i.e., more acetic acid is produced when the turbidity is lower) and pre-fermentation temperature (i.e., stronger effects at low temperature) [37]. In addition, the endogenous SO_2_ levels produced depend strongly on the yeast strain, availability of nutrients, such as nitrogen, pH, and temperature [58].

All of these studied strains produced low concentrations of acetic acid and H_2_S, which are positive characters in a fermenting strain. The most important factor in the selection of a yeast strain is the consumption of sugar, as the presence of residual sugar (i.e., >4 g/L) is indicative of incomplete fermentation [59]. The strains M.2.16, M.3.16, M.6.16, M.7.16, M.8.16, M.9.16, M.10.16, M.3.17, M.4.17, M.7.17, M.10.17 and M.11.17 almost entirely consumed the sugars during the alcoholic fermentation and showed acceptable alcohol yields. For these strains, the selection was based on the fermentation vigour and kinetics.

## 5. Conclusions

The exploitation of the biodiversity of indigenous strains has great importance for the characterisation and selection of strains with particular phenotypes, and for biodiversity preservation and exploitation in terms of the Lebanese culture collection of *S. cerevisiae* strains, which can be certified following international standards. The selection of native yeast can provide strains that are characterised by oenological traits different from those of the starter strains commercially available, such as reduced alcohol levels and the increased production of secondary metabolites. For this reason, as a result of this study, three wine yeast strains from this large representative population, namely M.6.16, M.10.16 and M.4.17, may be used as starters based on their favourable oenological properties: high fermentation vigour, low production of volatile acidity and H_2_S and low residual sugar content at the end of alcoholic fermentation. Alone or together, the selected strains appear to be promising for use at the winery scale to produce different types of ‘Merwah’ wine.

## Figures and Tables

**Table 1:** Characteristics of the 12 microsatellite loci for the *Saccharomyces cerevisiae* genotyping.

Mix	Site Name	Multiplexed Primers	Motif and Type	Fluorescent Dye	Reference
#1	C5-F	TGACACAATAGCAATGGCCTTCA	GT	5′-YAKYE	[22]
	C5-R	GCAAGCGACTAGAACAACAATCACA			
	SCYOR267C-F	TACTAACGTCAACACTGCTGCCAA	TGT	5′-YAKYE	[23,24]
	SCYOR267C-R	GGATCTACTTGCAGTATACGGG			
	C8-F	CAGGTCGTTCTAACGTTGGTAAAATG	TAA	5′-FAM	[22]
	C8-R	GCTGTTGCTGTTGGTAGCATTACTGT			
	C11-F	TTCCATCATAACCGTCTGGGATT	GT	5′-FAM	[25]
	C11-R	TGCCTTTTTCTTAGATGGGCTTTC			
	SCAAT2-F	CAGTCTTATTGCCTTGAACGA	TAA	5′-AT565	[24]
	SCAAT2-R	GTCTCCATCCTCCAAACAGCC			
#2	C9-F	AAGGGTTCGTAAACATATAACTGGCA	TAA	5′-AT550	[22]
	C9-R	TATAAGGGAAAAGAGCACGATGGC			
	C4-F	AGGAGAAAAATGCTGTTTATTCTGACC	TAA + TAG	5′-AT550	[22]
	C4-R	TTTTCCTCCGGGACGTGAAATA			
	SCAAT5-F	AGCATAATTGGAGGCAGTAAAGCA	TAA	5′-AT550	[22]
	SCAAT5-R	TCTCCGTCTTTTTTGTACTGCGTG			
	SCAAT1-F	AAAGCGTAAGCAATGGTGTAGATACTT	TTA	5′-YAKYE	[22,23,24]
	SCAAT1-R	CAAGCCTCTTCAAGCATGACCTTT			
	C6-F	GTGGCATCATATCTGTCAATTTTATCAC	CA	5′-YAKYE	[22]
	C6-R	CAATCAAGCAAAAGATCGGCCT			
	YKL172W-F	CAGGACGCTACCGAAGCTCAAAAG	GAA	5′-FAM	[25]
	YKL172W-R	ACTTTTGGCCAATTTCTCAAGAT			
	YPL009c-F	AACCCATTGACCTCGTTACTATCGT	CTT	5′-FAM	[23,24]
	YPL009c-R	TTCGATGGCTCTGATAACTCCATTC			

F, forward; R, reverse. YAKYE, Yakima yellow; FAM, carboxyfluorescein green; AT550, Cyanin bleu; AT565, Phycoerythrin (PE) red.

**Table 2:** Description of the 22 Lebanese indigenous *S. cerevisiae* strains used for the technological characterisation. EF = end of alcoholic fermentation.

Sample Code	Sample Name	Isolate Number	Harvesting Year
M.1.16	2016-1EF	3	2016
M.2.16	2016-1EF	16	2016
M.3.16	2016-1EF	6	2016
M.4.16	2016-2EF	1	2016
M.5.16	2016-1EF	2	2016
M.6.16	2016-2EF	3	2016
M.7.16	2016-2EF	5	2016
M.8.16	2016-2EF	8	2016
M.9.16	2016-3EF	9	2016
M.10.16	2016-3EF	6	2016
M.1.17	2017-1EF	3	2017
M.2.17	2017-1EF	20	2017
M.3.17	2017-1EF	21	2017
M.4.17	2017-1EF	39	2017
M.5.17	2017-1EF	49	2017
M.6.17	2017-1EF	54	2017
M.7.17	2017-1EF	66	2017
M.8.17	2017-2EF	4	2017
M.9.17	2017-2EF	5	2017
M.10.17	2017-2EF	9	2017
M.11.17	2017-2EF	16	2017
M.12.17	2017-2EF	17	2017

**Table 3:** Sample repartition and chemical analysis of the ‘Merwah’ samples collected at the middle and the end of the alcoholic fermentations over the two harvest seasons (2016, 2017).

Sample Name *	Vineyard Location	Number of Isolates	Mean Fermentation Temperature (°C)	Density	Sugar Concentration (g/L)	pH	Total Acidity (g/L H_2_SO_4_)
2016-1MF	Wata el Jozz	25	17	1.038	95	3.20	4.12
2016-1EF	Wata el Jozz	19	17	0.998	8	3.28	4.31
2016-2MF	Bekaatet Achout	40	17	1.038	103	3.28	3.62
2016-2EF	Bekaatet Achout	12	17	0.998	8	3.29	3.32
2016-3MF	Wata el Jozz Bekaatet Achout	1	17	1.040	96	3.32	3.68
2016-3EF	Wata el Jozz Bekaatet Achout	15	17	0.994	4	3.13	3.23
2017-1EF	Wata el Jozz Bekaatet Achout	71	18	0.996	8	3.15	4.21
2017-2EF	Wata el Jozz Bekaatet Achout	19	16	0.994	4	3.29	4.26

* The sample names refer to the year of the yeast isolation from the must (2016, 2017), the number of the batch (1, 2, 3), which differs according to vineyard location and grape maturity, and the stage of the alcoholic fermentation (MF, middle fermentation; EF, end fermentation).

**Table 4:** Pairwise fixation indices calculated using the distance matrix.

Population	Fixation Index (*Fst*) According to Yeast Strain Population			
Bioprocess	NA	0.056	0.026	0.017
Wild	0.056	NA	0.041	0.028
Wine (industrial)	0.026	0.041	NA	0.100
Lebanon ‘Merwah’ wine	0.017	0.028	0.100	NA

*Fst* <0.05, no genetic differentiation; 0.05 < *Fst* < 0.15, moderate genetic differentiation; 0.15 < *Fst* < 0.25, important genetic differentiation; *Fst* >0.25, high genetic differentiation [44]. NA, not applicable.

## Supplementary Materials

The following are available online at https://www.mdpi.com/2076-2607/7/11/492/s1. Table S1: Clustering data for the 202 ‘Merwah’ yeast strains isolated in 2016 and 2017 based on interdelta-PCR analysis, Table S2: Statistical analysis of the interdelta-PCR data for the 112 ‘Merwah’ wine yeast isolates from 2016, Table S3: Statistical analysis of the interdelta-PCR data for the 90 ‘Merwah’ wine yeast isolates from 2017, Table S4: *Saccharomyces cerevisiae* strains of different origins (138) added to the ‘Merwah’ wine yeast population in the microsatellite analysis, Table S5: Statistical differences in total CO2 production of 22 *S. cerevisiae* strains as determined by ANOVA followed by Tukey -HSD test (p<0.01). Strain showing not significant differences share common group letters, Table S6: Technological parameters of the 22 *Saccharomyces cerevisiae* strains during fermentation of the synthetic must.
