# Supplementary material for: Genetic and Phenotypic Characterisation of a Saccharomyces cerevisiae Population of ‘Merwah’ White Wine
Source: Microorganisms. 2019 Oct 26;7(11):492. doi: 10.3390/microorganisms7110492 (PMC6920927; doi:10.3390/microorganisms7110492)
Supplement: Supplementary file 1 [file microorganisms-07-00492-s001.pdf]

microorganisms

Supplementary Materials

Table S1. Clustering data for the 202 'Merwah' yeast strains isolated in 2016 and 2017, based on interdelta-PCR analysis.

Sample	Isolate	Observation Number	Cluster Number
2016-1MF	1	Obs1	C1
2016-1MF	2	Obs2	C1
2016-1MF	3	Obs3	C1
2016-1MF	4	Obs4	C1
2016-1MF	5	Obs5	C1
2016-1MF	6	Obs6	C1
2016-1MF	7	Obs7	C1
2016-1MF	8	Obs8	C1
2016-1MF	9	Obs9	C1
2016-1MF	10	Obs10	C1
2016-1MF	11	Obs11	C1
2016-1MF	12	Obs12	C1
2016-1MF	13	Obs13	C1
2016-1MF	14	Obs14	C1
2016-1MF	15	Obs15	C1
2016-1MF	16	Obs16	C1
2016-1MF	17	Obs17	C1
2016-1MF	18	Obs18	C1
2016-1MF	19	Obs19	C1
2016-1MF	20	Obs20	C1
2016-1MF	21	Obs21	C1
2016-1MF	22	Obs22	C1
2016-1MF	23	Obs23	C1
2016-1MF	24	Obs24	C1
2016-1MF	25	Obs25	C1
2016-1EF	1	Obs26	C1
2016-1EF	2	Obs27	C1
2016-1EF	3	Obs28	C1
2016-1EF	4	Obs29	C1
2016-1EF	5	Obs30	C1
2016-1EF	6	Obs31	C1
2016-1EF	7	Obs32	C1
2016-1EF	8	Obs33	C1
2016-1EF	9	Obs34	C1
2016-1EF	10	Obs35	C1
2016-1EF	11	Obs36	C1
2016-1EF	12	Obs37	C1
2016-1EF	13	Obs38	C1
2016-1EF	14	Obs39	C1
2016-1EF	15	Obs40	C1
2016-1EF	16	Obs41	C1
2016-1EF	17	Obs42	C1
2016-1EF	18	Obs43	C1
2016-1EF	19	Obs44	C1

2016-2MF	1	Obs45	C1
2016-2MF	2	Obs46	C1
2016-2MF	3	Obs47	C1
2016-2MF	4	Obs48	C1
2016-2MF	5	Obs49	C1
2016-2MF	6	Obs50	C1
2016-2MF	7	Obs51	C1
2016-2MF	8	Obs52	C1
2016-2MF	9	Obs53	C1
2016-2MF	10	Obs54	C1
2016-2MF	11	Obs55	C1
2016-2MF	12	Obs56	C1
2016-2MF	13	Obs57	C1
2016-2MF	14	Obs58	C1
2016-2MF	15	Obs59	C1
2016-2MF	16	Obs60	C1
2016-2MF	17	Obs61	C1
2016-2MF	18	Obs62	C1
2016-2MF	19	Obs63	C1
2016-2MF	20	Obs64	C1
2016-2MF	21	Obs65	C1
2016-2MF	22	Obs66	C1
2016-2MF	23	Obs67	C1
2016-2MF	24	Obs68	C1
2016-2MF	25	Obs69	C1
2016-2MF	26	Obs70	C1
2016-2MF	27	Obs71	C1
2016-2MF	28	Obs72	C1
2016-2MF	29	Obs73	C1
2016-2MF	30	Obs74	C1
2016-2MF	31	Obs75	C1
2016-2MF	32	Obs76	C1
2016-2MF	33	Obs77	C1
2016-2MF	34	Obs78	C1
2016-2MF	35	Obs79	C1
2016-2MF	36	Obs80	C1
2016-2MF	37	Obs81	C1
2016-2MF	38	Obs82	C1
2016-2MF	39	Obs83	C1
2016-2MF	40	Obs84	C1
2016-2EF	1	Obs85	C1
2016-2EF	2	Obs86	C1
2016-2EF	3	Obs87	C1
2016-2EF	4	Obs88	C1
2016-2EF	5	Obs89	C1
2016-2EF	6	Obs90	C1
2016-2EF	7	Obs91	C1
2016-2EF	8	Obs92	C1
2016-2EF	9	Obs93	C1
2016-2EF	10	Obs94	C1
2016-2EF	11	Obs95	C2

2016-2EF	12	Obs96	C2
2016-3MF	1	Obs97	C2
2016-3EF	2	Obs98	C2
2016-3EF	3	Obs99	C2
2016-3EF	4	Obs100	C2
2016-3EF	5	Obs101	C2
2016-3EF	6	Obs102	C2
2016-3EF	7	Obs103	C2
2016-3EF	8	Obs104	C2
2016-3EF	9	Obs105	C2
2016-3EF	10	Obs106	C2
2016-3EF	11	Obs107	C2
2016-3EF	12	Obs108	C2
2016-3EF	13	Obs109	C2
2016-3EF	14	Obs110	C2
2016-3EF	15	Obs111	C2
2016-3EF	16	Obs112	C2
2017-1EF	1	Obs113	C2
2017-1EF	2	Obs114	C2
2017-1EF	3	Obs115	C2
2017-1EF	4	Obs116	C2
2017-1EF	5	Obs117	C2
2017-1EF	6	Obs118	C2
2017-1EF	7	Obs119	C2
2017-1EF	8	Obs120	C2
2017-1EF	9	Obs121	C2
2017-1EF	10	Obs122	C2
2017-1EF	11	Obs123	C2
2017-1EF	12	Obs124	C2
2017-1EF	13	Obs125	C2
2017-1EF	14	Obs126	C2
2017-1EF	15	Obs127	C2
2017-1EF	16	Obs128	C2
2017-1EF	17	Obs129	C2
2017-1EF	18	Obs130	C2
2017-1EF	19	Obs131	C2
2017-1EF	20	Obs132	C2
2017-1EF	21	Obs133	C2
2017-1EF	22	Obs134	C2
2017-1EF	23	Obs135	C2
2017-1EF	24	Obs136	C2
2017-1EF	25	Obs137	C2
2017-1EF	26	Obs138	C2
2017-1EF	27	Obs139	C2
2017-1EF	28	Obs140	C2
2017-1EF	29	Obs141	C2
2017-1EF	30	Obs142	C2
2017-1EF	31	Obs143	C2
2017-1EF	32	Obs144	C2
2017-1EF	33	Obs145	C2
2017-1EF	34	Obs146	C2

2017-1EF	35	Obs147	C2
2017-1EF	36	Obs148	C2
2017-1EF	37	Obs149	C2
2017-1EF	38	Obs150	C2
2017-1EF	39	Obs151	C2
2017-1EF	40	Obs152	C2
2017-1EF	41	Obs153	C2
2017-1EF	42	Obs154	C2
2017-1EF	43	Obs155	C2
2017-1EF	44	Obs156	C2
2017-1EF	45	Obs157	C2
2017-1EF	46	Obs158	C2
2017-1EF	47	Obs159	C2
2017-1EF	48	Obs160	C2
2017-1EF	49	Obs161	C2
2017-1EF	50	Obs162	C2
2017-1EF	51	Obs163	C2
2017-1EF	52	Obs164	C2
2017-1EF	53	Obs165	C2
2017-1EF	54	Obs166	C3
2017-1EF	55	Obs167	C3
2017-1EF	56	Obs168	C3
2017-1EF	57	Obs169	C3
2017-1EF	58	Obs170	C3
2017-1EF	59	Obs171	C3
2017-1EF	60	Obs172	C3
2017-1EF	61	Obs173	C3
2017-1EF	62	Obs174	C3
2017-1EF	63	Obs175	C3
2017-1EF	64	Obs176	C3
2017-1EF	65	Obs177	C3
2017-1EF	66	Obs178	C3
2017-1EF	67	Obs179	C3
2017-1EF	68	Obs180	C3
2017-1EF	69	Obs181	C3
2017-1EF	70	Obs182	C3
2017-1EF	71	Obs183	C3
2017-2EF	1	Obs184	C3
2017-2EF	2	Obs185	C3
2017-2EF	3	Obs186	C3
2017-2EF	4	Obs187	C3
2017-2EF	5	Obs188	C3
2017-2EF	6	Obs189	C3
2017-2EF	7	Obs190	C3
2017-2EF	8	Obs191	C3
2017-2EF	9	Obs192	C3
2017-2EF	10	Obs193	C3
2017-2EF	11	Obs194	C3
2017-2EF	12	Obs195	C3
2017-2EF	13	Obs196	C3
2017-2EF	14	Obs197	C3

2017-2EF	15	Obs198	C3
2017-2EF	16	Obs199	C3
2017-2EF	17	Obs200	C3
2017-2EF	18	Obs201	C3
2017-2EF	19	Obs202	C3

A significant cluster difference that splits 2016 from 2017. This dendrogram regroup the isolates into 3 clusters as following: C1 = 112 observations from Merwah 2016 + 3 observations from Merwah 2017; C2 = 50 observations from Merwah 2017; C3 = 37 observations from Merwah 2017.

Table S2. Statistical analysis of the interdelta-PCR data for the 112 'Merwah' wine yeast isolates from 2016.

Sample Name	Observation Number	Cluster Number
2016-1MF	1	C1
2016-1MF	2	C1
2016-1MF	3	C1
2016-1MF	4	C1
2016-1MF	5	C1
2016-1MF	6	C1
2016-1MF	7	C1
2016-1MF	8	C1
2016-1MF	9	C1
2016-1MF	10	C1
2016-1MF	11	C1
2016-1MF	12	C1
2016-1MF	13	C1
2016-1MF	14	C1
2016-1MF	15	C1
2016-1MF	16	C1
2016-1MF	17	C1
2016-1MF	18	C1
2016-1MF	19	C1
2016-1MF	20	C1
2016-1MF	21	C1
2016-1MF	25	C1
2016-1EF	26	C1
2016-1EF	28	C1
2016-1EF	29	C1
2016-1EF	30	C1
2016-1EF	31	C1
2016-1EF	32	C1
2016-1EF	33	C1
2016-1EF	34	C1
2016-1EF	35	C1
2016-1EF	36	C1
2016-1EF	37	C1
2016-1EF	38	C1
2016-1EF	39	C1
2016-1EF	41	C1
2016-2MF	45	C1
2016-2MF	46	C1
2016-2MF	47	C1

2016-2MF	48	C1
2016-2MF	49	C1
2016-2MF	50	C1
2016-2MF	51	C1
2016-2MF	52	C1
2016-2MF	53	C1
2016-2MF	54	C1
2016-2MF	55	C1
2016-2MF	56	C1
2016-2MF	57	C1
2016-2MF	58	C1
2016-2MF	59	C1
2016-2MF	60	C1
2016-2MF	61	C1
2016-2MF	62	C1
2016-2MF	63	C1
2016-2MF	64	C1
2016-2MF	65	C1
2016-2MF	66	C1
2016-2MF	67	C1
2016-2MF	68	C1
2016-2MF	69	C1
2016-2MF	70	C1
2016-2MF	71	C1
2016-2MF	72	C1
2016-2MF	73	C1
2016-2MF	74	C1
2016-2MF	75	C1
2016-2MF	76	C1
2016-2MF	77	C1
2016-2MF	78	C1
2016-2MF	79	C1
2016-2MF	80	C1
2016-2MF	81	C1
2016-2MF	82	C1
2016-2MF	83	C1
2016-2MF	84	C1
2016-2EF	85	C1
2016-2EF	86	C1
2016-2EF	87	C1
2016-2EF	88	C1
2016-2EF	89	C1
2016-2EF	90	C1
2016-2EF	91	C1
2016-2EF	92	C1
2016-2EF	93	C1
2016-2EF	94	C1
2016-2EF	95	C1
2016-2EF	96	C1
2016-3MF	97	C1
2016-3EF	98	C1

2016-3EF	99	C1
2016-3EF	100	C1
2016-3EF	101	C1
2016-3EF	102	C1
2016-3EF	103	C1
2016-3EF	104	C1
2016-3EF	105	C1
2016-3EF	106	C1
2016-3EF	107	C1
2016-3EF	108	C1
2016-3EF	109	C1
2016-3EF	110	C1
2016-3EF	111	C1
2016-3EF	112	C1
2016-1MF	22	C2
2016-1MF	23	C2
2016-1MF	24	C2
2016-1EF	27	C3
2016-1EF	43	C3
2016-1EF	40	C4
2016-1EF	42	C4
2016-1EF	44	C4

Table S3. Statistical analysis of the interdelta-PCR data for the 90 'Merwah' wine yeast isolates from 2017.

Sample Name	Observation Number	Cluster Number
2017-1EF	1	C1
2017-1EF	3	C1
2017-1EF	23	C1
2017-1EF	54	C1
2017-1EF	59	C1
2017-1EF	61	C1
2017-2EF	75	C1
2017-2EF	84	C1
2017-2EF	87	C1
2017-1EF	2	C2
2017-1EF	4	C2
2017-1EF	6	C2
2017-1EF	7	C2
2017-1EF	8	C2
2017-1EF	9	C2
2017-1EF	10	C2
2017-1EF	11	C2
2017-1EF	12	C2
2017-1EF	13	C2
2017-1EF	14	C2
2017-1EF	16	C2
2017-1EF	17	C2
2017-1EF	18	C2
2017-1EF	19	C2
2017-1EF	20	C2

2017-1EF	21	C2
2017-1EF	22	C2
2017-1EF	24	C2
2017-1EF	25	C2
2017-1EF	26	C2
2017-1EF	27	C2
2017-1EF	29	C2
2017-1EF	30	C2
2017-1EF	31	C2
2017-1EF	32	C2
2017-1EF	33	C2
2017-1EF	34	C2
2017-1EF	36	C2
2017-1EF	37	C2
2017-1EF	38	C2
2017-1EF	40	C2
2017-1EF	42	C2
2017-1EF	44	C2
2017-1EF	45	C2
2017-1EF	47	C2
2017-1EF	48	C2
2017-1EF	49	C2
2017-1EF	50	C2
2017-1EF	51	C2
2017-1EF	52	C2
2017-1EF	53	C2
2017-1EF	55	C2
2017-1EF	56	C2
2017-1EF	57	C2
2017-1EF	58	C2
2017-1EF	60	C2
2017-1EF	62	C2
2017-1EF	63	C2
2017-1EF	65	C2
2017-1EF	67	C2
2017-1EF	68	C2
2017-1EF	69	C2
2017-1EF	70	C2
2017-2EF	72	C2
2017-2EF	73	C2
2017-2EF	76	C2
2017-2EF	77	C2
2017-2EF	78	C2
2017-2EF	79	C2
2017-2EF	80	C2
2017-2EF	81	C2
2017-2EF	82	C2
2017-2EF	90	C2
2017-1EF	5	C3
2017-1EF	28	C3
2017-1EF	35	C3

2017-1EF	39	C3
2017-1EF	46	C3
2017-1EF	71	C3
2017-2EF	74	C3
2017-2EF	83	C3
2017-2EF	89	C3
2017-1EF	15	C4
2017-1EF	41	C4
2017-1EF	43	C4
2017-1EF	64	C4
2017-1EF	66	C4
2017-2EF	85	C4
2017-2EF	86	C4
2017-2EF	88	C4

Table S4. *Saccharomyces cerevisiae* strains of different origins added to the 'Merwah' wine yeast population in the microsatellite analysis.

Strain Name	Strain Type	Origin	Country
YS2	Bioprocess	Bread	Australia
YS4	Bioprocess	Bread	The Netherlands
YS9	Bioprocess	Bread	Singapore
Y9	Bioprocess	Sake	Indonesia
Y12	Bioprocess	Palm wine	Ivory Coast
NCYC110	Bioprocess	Beer	West Africa
K11	Bioprocess	Sake	Japan
DBVP66044	Bioprocess	Beer	West Africa
223R	Wine	Industrial	NR
Actiflore 522	Wine	Industrial	NR
Actiflore BJL	Wine	Industrial	NR
Actiflore BO213	Wine	Industrial	NR
Actiflore F33	Wine	Industrial	NR
Actiflore ROSE	Wine	Industrial	NR
Affinity ECAS	Wine	Industrial	NR
ANCHOR ALCHEMY	Wine	Industrial	NR
ANCHOR EXOTICS SPH	Wine	Industrial	NR
ANCHOR NT116	Wine	Industrial	NR
ANCHOR NT202	Wine	Industrial	NR
ANCHOR NT45	Wine	Industrial	NR
ANCHOR NT50	Wine	Industrial	NR
ANCHOR VIN13	Wine	Industrial	NR
C17	Wine	Industrial	NR
COGNAC-7103	Wine	Industrial	NR
Collection cepage sauvignon	Wine	Industrial	NR
E2F	Wine	Industrial	NR
EQUINOX-81	Wine	Industrial	NR
EXCELLENCE SP	Wine	Industrial	NR
EXCELLENCE 8S	Wine	Industrial	NR
EXCELLENCE STR	Wine	Industrial	NR
EXCELLENCE TXL	Wine	Industrial	NR
EXCELLENCE XR	Wine	Industrial	NR
FC9	Wine	Industrial	NR
FERMICHAMP	Wine	Industrial	NR
FERMOL CANDY	Wine	Industrial	NR
FERMOL Cryofruit	Wine	Industrial	NR
FERMOL IPER-R	Wine	Industrial	NR
FERMOL PB2033	Wine	Industrial	NR
FERMOL PRIMEURS	Wine	Industrial	NR
FERMOL RED FRUIT	Wine	Industrial	NR
FERMOL SPIRIT	Wine	Industrial	NR

H4	Wine	Industrial	NR
ICV D254	Wine	Industrial	NR
ICVOK	Wine	Industrial	NR
IOC18-2007	Wine	Industrial	NR
I onis	Wine	Industrial	NR
LA-PM	Wine	Industrial	NR
LALVIN CLOS	Wine	Industrial	NR
LALVIN FC9 EDV	Wine	Industrial	NR
LALVIN QA23	Wine	Industrial	NR
LALVIN RC 212	Wine	Industrial	NR
LALVIN RHONE 2056	Wine	Industrial	NR
LALVIN RHONE 2226	Wine	Industrial	NR
LEP 55	Wine	Industrial	NR
LEVULIA ESPERIDE	Wine	Industrial	NR
LEVULIA GE7 TRADITION	Wine	Industrial	NR
LEVULIA U 32	Wine	Industrial	NR
LEVULINE CHP	Wine	Industrial	NR
MAURIVIN AWRI 350	Wine	Industrial	NR
MAURIVIN UOA MAXI	Wine	Industrial	NR
NT 202	Wine	Industrial	NR
NT 50	Wine	Industrial	NR
PREDELVIN PDM	Wine	Industrial	NR
RB 2	Wine	Industrial	NR
RB 4	Wine	Industrial	NR
RMS 2	Wine	Industrial	NR
RX 60	Wine	Industrial	NR
SAFOENO HD S62	Wine	Industrial	NR
SAFOENO UVA S111	Wine	Industrial	NR
SAUVIGNON	Wine	Industrial	NR
SELECTYS L-AUTHENT	Wine	Industrial	NR
SELECTYS L-ELEGANTE	Wine	Industrial	NR
SELECTYS LA PERSANE	Wine	Industrial	NR
SELECTYS LA RAFFINEE	Wine	Industrial	NR
SELECTYS SR	Wine	Industrial	NR
SO DELIGHT	Wine	Industrial	NR
SO FLAVOUR	Wine	Industrial	NR
SO SPIRIT	Wine	Industrial	NR
SP 39	Wine	Industrial	NR
SP 49	Wine	Industrial	NR
SPARKLSA	Wine	Industrial	NR
SPINGER VR 44	Wine	Industrial	NR
SPINGER BC S 103	Wine	Industrial	NR
SPINGER CK S 102	Wine	Industrial	NR
SPINGER NDA 21	Wine	Industrial	NR
SPINGER SAINT GEORGES-S 101	Wine	Industrial	NR
SPINGER UCLM S 325	Wine	Industrial	NR
SPINGER UCLM S 377	Wine	Industrial	NR
VIALATTE FERM R 100	Wine	Industrial	NR
VIALATTE FERM R 71	Wine	Industrial	NR
VIALATTE FERM R 82	Wine	Industrial	NR
VIALATTE FERM R 96	Wine	Industrial	NR
VIALATTE FERM W 12	Wine	Industrial	NR
VIALETTE FERM W 28	Wine	Industrial	NR
VINIFERM CT 007	Wine	Industrial	NR
VINIFERM EMOCION	Wine	Industrial	NR
VINIFERM RVA	Wine	Industrial	NR
VINIFLORA MELODY	Wine	Industrial	NR
VINIFLORA MERIT	Wine	Industrial	NR
VITILEVURE 3001	Wine	Industrial	NR
VITILEVURE DV10	Wine	Industrial	NR
VITILEVURE ELXIR	Wine	Industrial	NR
VITILEVURE QUARTZ	Wine	Industrial	NR
Yseo brio	Wine	Industrial	NR
ZYMAFLORE CH09	Wine	Industrial	NR

ZYMAFLORE DELTA	Wine	Industrial	NR
ZYMAFLORE F15	Wine	Industrial	NR
ZYMAFLORE FX10	Wine	Industrial	NR
ZYMAFLORE ST	Wine	Industrial	NR
ZYMAFLORE VL1	Wine	Industrial	NR
ZYMAFLORE VL2	Wine	Industrial	NR
ZYMAFLORE VL3	Wine	Industrial	NR
ZYMAFLORE XS	Wine	Industrial	NR
ZYMAFLORE XPURE	Wine	Industrial	NR
ZYMAZIL	Wine	Industrial	NR
BC 187	Wine	Wine	USA
DBVPG1106	Wine	Grape	Australia
273614 X	Wild	Clinical	UK
378604 X	Wild	Clinical	UK
DBVPG 1853	Wild	Cereal	Ethiopia
DBVPG 6765	Wild	Fruit	Indonesia
L-1374	Wine	Wine	Chile
L-1528	Wine	Wine	Chile
S288c	Wild	Fruit	USA
SK1	Wild	Soil	USA
UWOPSO3	Wild	Plant	Malaysia
UWOPSO5.2	Wild	Insect	Malaysia
UWOPS83	Wild	Fruit	Bahamas
UWOPS87	Wild	Plant	Hawai
W303	NA	Laboratory	NA
Y 55	Wine	Grape	France
YII C17 E5	Wine	Wine	France
YJM 975	Wild	Clinical	Italy
YJM 978	Wild	Clinical	Italy
YJM 981	Wild	Clinical	Italy
YPS 606	Wild	Plant	USA

NR, not recorded; NA, not applicable.

Table S5. Technological parameters of the 22 *Saccharomyces cerevisiae* strains during fermentation of the synthetic grape juice.

Sample Name	Fermentation Time (days)	Fermentation Vigour (g/100mL)	CO_2 Production (g/100 mL)	H_2S Production	Free SO_2 (ppm)	Total SO_2 (ppm)	Total Acidity (g/L Sulfuric Acid)	Volatile Acidity (g/L Acetic Acid)	pH	Residual Sugar (g/L)	Ethanol Production (%)
M.1.16	14	0.95±0.02	7.17±0.21	1	3.40±0.02	0	3.6 ±0.24	0.69 ±0.23	3.16 ±0.01	3.83 ±0.36	11.66 ±0.02
M.2.16	13	1.45±0.16	10.40±0.43	3	4.20±0.03	0	3.8 ±0.05	0.02±0.002	3.2 ±0.03	0.94 ±0.04	11.83 ±0.02
M.3.16	11	2.95±0.18	10.37±0.24	1	5.50±0.02	34.50±0.02	3.5 ±0.4	0.50 ±0.06	3.265 ±0.05	0.12 ±0.04	11.88 ±0.003
M.4.16	13	1.32±0.01	4.03±0.05	2	4.80±0.05	23.50±0.07	3.56 ±0.23	0.55 ±0.24	3.18 ±0.08	8.04 ±1.35	11.41 ±0.08
M.5.16	12	1.68±0.07	6.02±0.06	2	5.30±0.04	32.00±0.03	4.0 ±0.06	0.52 ±0.04	3.39 ±0.06	4.54 ±0.43	11.61 ±0.03
M.6.16	12	3.40±0.22	10.05±0.24	1	5.30±0.04	19.50±0.02	4.7 ±0.17	0.53 ±0.004	3.54 ±0.04	0.40±0.07	11.86 ±0.004
M.7.16	11	1.45±0.01	7.12±0.06	2	5.80±0.13	42.00±0.14	3.9 ±0.3	0.31±0.025	3.4 ±0.041	1.90 ±0.22	11.77 ±0.01
M.8.16	10	1.68±0.06	9.08±0.02	1	4.95±0.03	40.00±0.36	3.75 ±0.25	0.31 ±0.043	3.39 ±0.03	0.91 ±0.02	11.83 ±0.01
M.9.16	9	3.20±0.22	9.40±0.22	2	3.9±0.23	8.00±0.03	4.8 ±0.08	0.33 ±0.081	3.23 ±0.12	0.45 ±0.06	11.86 ±0.03
M.10.16	10	3.53±0.21	9.02±0.09	1	0	0	3.97 ±0.16	0.021±0.09	3.40 ±0.16	2.3 ±0.242	11.75 ±0.01
M.1.17	8	2.60±0.37	4.78±0.17	2	5.00±0.14	0	4.6 ±0.59	0.32 ±0.003	3.17 ±0.23	7.6 ±0.864	11.43 ±0.05
M.2.17	9	2.97±0.62	7.56±0.29	1	4.90±0.02	1	4.2 ±0.03	0.02 ±0.005	3.23 ±0.05	3.06 ±0.03	11.70 ±0.002
M.3.17	10	3.27±0.05	8.23±027	2	3.60±0.05	5.00±0.10	3.3 ±0.04	0.05 ±0.01	3.53 ±0.06	0.94 ±0.02	11.83 ±0.001
M.4.17	10	3.20±0.25	9.27±0.22	1	0.23±0.03	0.53±0.03	3.5 ±0.06	0.06 ±0.002	3.21 ±0.13	0.74 ±0.03	11.84 ±0.002
M.5.17	11	0.80±0.29	3.18±1.08	2	0.33±0.20	0.60±0.01	3.62 ±0.23	0.34 ±0.048	3.36 ±0.06	13.6 ±1.33	11.07 ±0.19
M.6.17	10	1.90±0.43	6.60±0.67	1	0.13±0.04	0.53±0.16	3.35 ±0.15	0.24 ±0.10	3.19 ±0.02	3.96 ±1.76	11.65 ±0.105
M.7.17	14	1.44±0.06	8.33 ±1.25	3	0.47 ±0.06	0.38 ±0.18	3.78 ±0.46	0.04±0.02	3.37 ±0.60	1.07 ±0.16	11.82 ±0.01
M.8.17	11	1.87±0.13	7.23 ±0.35	1	0.38 ±0.07	0.41 ±0.03	4.10 ±0.22	0.01±0.002	3.61 ±0.04	4.30 ±0.32	11.63 ±0.02
M.9.17	11	1.3±0.31	5.97 ±0.22	2	0.5 ±0.023	0.65 ±0.03	3.36 ±0.07	0.15±0.009	3.51 ±0.17	4.85 ±0.25	11.60 ±0.015
M.10.17	13	1.51±0.19	8.95 ±0.79	1	0.53 ±0.003	0.80 ±0.12	3.58 ±0.05	0.13±0.002	3.42 ±0.38	0.39 ±0.17	11.86 ±0.01
M.11.17	15	1.72±0.11	9.68 ±0.16	1	0.64 ±0.002	0.93 ±0.02	3.84 ±0.06	0.17±0.03	3.26 ±0.06	0.63 ±0.09	11.85 ±0.006
M.12.17	15	1.67±0.09	7.89 ±0.23	1	0.65 ±0.03	0.60 ±0.04	4.27 ±0.18	0.07±0.009	3.50 ±0.05	3.45 ±0.11	11.68 ±0.007

Table S6. Technological parameters of the 22 Saccharomyces cerevisiae strains during fermentation of the synthetic must.

Sample Name	Fermentation Time (days)	Fermentation Vigour (g/100mL)	CO_2 Production (g/100 mL)	H2S Production	Free SO_2 (ppm)	Total SO_2 (ppm)	Total Acidity (g/L Sulfuric Acid)	Volatile Acidity (g/L Acetic Acid)	pH	Residual Sugar (g/L)	Ethanol Production (%)
M.1.16	14	0.95±0.02	7.17±0.21	1	3.40±0.02	0	3.6 ±0.24	0.69 ±0.23	3.16 ±0.01	3.83 ±0.36	11.66 ±0.02
M.2.16	13	1.45±0.16	10.40±0.43	3	4.20±0.03	0	3.8 ±0.05	0.02±0.002	3.2 ±0.03	0.94 ±0.04	11.83 ±0.02
M.3.16	11	2.95±0.18	10.37±0.24	1	5.50±0.02	34.50±0.02	3.5 ±0.4	0.50 ±0.06	3.26±0.05	0.12 ±0.04	11.88 ±0.003
M.4.16	13	1.32±0.01	4.03±0.05	2	4.80±0.05	23.50±0.07	3.56 ±0.23	0.55 ±0.24	3.18 ±0.08	8.04 ±1.35	11.41 ±0.08
M.5.16	12	1.68±0.07	6.02±0.06	2	5.30±0.04	32.00±0.03	4.0 ±0.06	0.52 ±0.04	3.39 ±0.06	4.54 ±0.43	11.61 ±0.03
M.6.16	12	3.40±0.22	10.05±0.24	1	5.30±0.04	19.50±0.02	4.7 ±0.17	0.53 ±0.004	3.54 ±0.04	0.40±0.07	11.86 ±0.004
M.7.16	11	1.45±0.01	7.12±0.06	2	5.80±0.13	42.00±0.14	3.9 ±0.3	0.31±0.025	3.4 ±0.041	1.90 ±0.22	11.77 ±0.01
M.8.16	10	1.68±0.06	9.08±0.02	1	4.95±0.03	40.00±0.36	3.75 ±0.25	0.31 ±0.043	3.39 ±0.03	0.91 ±0.02	11.83 ±0.01
M.9.16	9	3.20±0.22	9.40±0.22	2	3.9±0.23	8.00±0.03	4.8 ±0.08	0.33 ±0.081	3.23 ±0.12	0.45 ±0.06	11.86 ±0.03
M.10.16	10	3.53±0.21	9.02±0.09	1	0	0	3.97 ±0.16	0.021±0.09	3.40 ±0.16	2.3 ±0.242	11.75 ±0.01

M.1.17	8	2.60±0.37	4.78±0.17	2	5.00±0.14	0	4.6 ±0.59	0.32 ±0.003	3.17 ±0.23	7.6 ±0.864	11.43 ±0.05
M.2.17	9	2.97±0.62	7.56±0.29	1	4.90±0.02	1	4.2 ±0.03	0.02 ±0.005	3.23 ±0.05	3.06 ±0.03	11.70 ±0.002
M.3.17	10	3.27±0.05	8.23±027	2	3.60±0.05	5.00±0.10	3.3 ±0.04	0.05 ±0.01	3.53 ±0.06	0.94 ±0.02	11.83 ±0.001
M.4.17	10	3.20±0.25	9.27±0.22	1	0.23±0.03	0.53±0.03	3.5 ±0.06	0.06 ±0.002	3.21 ±0.13	0.74 ±0.03	11.84 ±0.002
M.5.17	11	0.80±0.29	3.18±1.08	2	0.33±0.20	0.60±0.01	3.62 ±0.23	0.34 ±0.048	3.36 ±0.06	13.6 ±1.33	11.07 ±0.19
M.6.17	10	1.90±0.43	6.60±0.67	1	0.13±0.04	0.53±0.16	3.35 ±0.15	0.24 ±0.10	3.19 ±0.02	3.96 ±1.76	11.65 ±0.105
M.7.17	14	1.44±0.06	8.33 ±1.25	3	0.47 ±0.06	0.38 ±0.18	3.78 ±0.46	0.04±0.02	3.37 ±0.60	1.07 ±0.16	11.82 ±0.01
M.8.17	11	1.87±0.13	7.23 ±0.35	1	0.38 ±0.07	0.41 ±0.03	4.10 ±0.22	0.01±0.002	3.61 ±0.04	4.30 ±0.32	11.63 ±0.02
M.9.17	11	1.3±0.31	5.97 ±0.22	2	0.5 ±0.023	0.65 ±0.03	3.36 ±0.07	0.15±0.009	3.51 ±0.17	4.85 ±0.25	11.60 ±0.015
M.10.17	13	1.51±0.19	8.95 ±0.79	1	0.53±0.003	0.80 ±0.12	3.58 ±0.05	0.13±0.002	3.42 ±0.38	0.39 ±0.17	11.86 ±0.01
M.11.17	15	1.72±0.11	9.68 ±0.16	1	0.64±0.002	0.93 ±0.02	3.84 ±0.06	0.17±0.03	3.26 ±0.06	0.63 ±0.09	11.85 ±0.006
M.12.17	15	1.67±0.09	7.89 ±0.23	1	0.65±0.03	0.60 ±0.04	4.27 ±0.18	0.07±0.009	3.50 ±0.05	3.45 ±0.11	11.68 ±0.007
